# Boric acid enhances the antitumor effects of paclitaxel by modulating apoptosis, autophagy-related protein expression, and proinflammatory cytokine expression in OVCAR-3 ovarian cancer cells

**DOI:** 10.55730/1300-0144.6377

**Published:** 2026-06-18

**Authors:** Nazmiye BİTGEN, Gözde Özge ÖNDER, Münevver BARAN, Fatma Esen KARAKUŞ, Melike AKBULUT, Arzu YAY

**Affiliations:** 1 Department of Medical Biology, Faculty of Medicine, Erciyes University, Kayseri, Turkiye; 2 Genome and Stem Cell Center (GENKOK), Erciyes University, Kayseri, Turkiye; 3 Department of Histology and Embryology, Faculty of Medicine, Erciyes University, Kayseri, Turkiye; 4 Department of Basic Science, Faculty of Pharmacy, Erciyes University, Kayseri, Turkiye; 5 Department of Medical Biology, Institute of Health Sciences, Erciyes University, Kayseri, Turkiye; 6 Department of Molecular Biology and Genetics, Gevher Nesibe Genome and Stem Cell Institute, Erciyes University, Kayseri, Turkiye

**Keywords:** Boric acid, apoptosis, autophagy, inflammation, OVCAR-3

## Abstract

**Background/aim:**

Ovarian cancer is the eighth most prevalent malignancy among women globally and ranks third among gynecological cancers in terms of incidence. The limited efficacy of standard chemotherapy, primarily due to intrinsic drug resistance and high recurrence rates, highlights the urgent need for novel therapeutic strategies that are both effective and selectively cytotoxic.

**Materials and methods:**

This study aimed to investigate the combined effects of boric acid (BA), a biologically active boron compound with emerging antitumor potential, and paclitaxel (PTX), a conventional chemotherapeutic agent, on cell viability, cell cycle progression, apoptosis, autophagy-related protein expression, and inflammatory signaling in the chemoresistant OVCAR-3 ovarian cancer cell line. OVCAR-3 cells were treated with increasing concentrations of BA over varying time periods. The half-maximal inhibitory concentrations (IC_50_) of both BA and PTX were determined using the MTT assay. Flow cytometry was employed to assess cell cycle distribution and apoptosis via Annexin V staining. Additionally, immunofluorescence staining was performed to evaluate the expression of autophagy-related proteins (Beclin-1, LC3, and p62) and proinflammatory cytokines (TNF-α, IL-6, and IL-1β).

**Results:**

The combination of BA and PTX resulted in significant G_0_/G_1_ phase cell cycle arrest and a marked increase in apoptotic cell death and the expression of autophagy-related proteins, leading to reduced cell viability in OVCAR-3 cells. Furthermore, cotreatment significantly modulated the expression of proinflammatory cytokines, suggesting a potential immunomodulatory effect associated with the observed cytotoxicity.

**Conclusion:**

The findings of this study provide evidence that boric acid enhances the antitumor efficacy of paclitaxel in drug-resistant ovarian cancer cells through coordinated modulation of cell cycle progression, apoptosis, autophagy-related protein expression, and inflammatory signaling. These findings support the potential of BA as a promising adjuvant for combination chemotherapy in ovarian cancer.

## 1. Introduction

Worldwide, ovarian cancer is considered one of the most aggressive malignancies of the female reproductive system. In 2023 alone, there were about 20,000 new cases and over 13,000 deaths in the US [1]. This disease is characterized by the uncontrolled proliferation of ovarian epithelial cells. Although patients continue to benefit from conventional treatment modalities, including surgery and chemotherapy, the 5-year survival rate remains unacceptably low because of the high recurrence rate, with relapse typically occurring within 6–12 months after chemotherapy [2]. A large number of women are diagnosed in the later stages of the disease (FIGO stage III or IV), which greatly worsens the prognosis [3]. To address this challenge, alternative strategies, including combination and sequential chemotherapy, have been proposed to improve therapeutic efficacy [4]. In addition, it is crucial to develop reliable preclinical experimental models of human ovarian cancer biology [5]. The OVCAR-3 cell line, which was established from a human ovarian adenocarcinoma, is well known for its chemoresistance and metastatic potential, making it a widely used model for investigating cellular responses to anticancer therapies [6,7]. Paclitaxel (PTX), a microtubule-stabilizing agent, is one of the cornerstone chemotherapeutic agents used in the treatment of ovarian cancer. Its antitumor activity is the result of disrupting microtubule turnover and causing cells to be arrested in mitosis. However, the development of resistance to PTX often results in therapeutic failure [8].

The trace element boron (B) is considered minimally toxic to mammals; however, it has attracted considerable interest because of its potential biomedical applications [9,10]. The major form of boron in the human body is boric acid (BA) [11]. BA has been shown to exert antiproliferative effects in a variety of cancer models, including lung [12], cervical [13], and prostate cancers [14,15]. Additionally, BA has been documented to induce apoptosis in melanoma and breast cancer cell lines [16,17]. Although boric acid exhibits promising anticancer and antiinflammatory properties, excessive exposure may lead to adverse effects. High-dose animal studies have reported reproductive and developmental toxicity, particularly affecting male fertility and fetal development, whereas human studies have not consistently demonstrated comparable effects. Therefore, careful dose optimization remains essential for potential therapeutic applications [18]. Despite these findings, the biological mechanisms underlying the effects of BA remain incompletely understood. Because boric acid has been reported to modulate several of these biological pathways, it may enhance the response of chemoresistant ovarian cancer cells to paclitaxel treatment. Therefore, this study aimed to investigate the cytotoxic, apoptotic, autophagy-related, and proinflammatory effects of BA and PTX, administered individually and in combination, in the OVCAR-3 ovarian cancer and human umbilical vein endothelial cell lines (HUVEC).

## 2. Materials and methods

### 2.1. Cell lines and culture conditions

The chemoresistant epithelial ovarian cancer cell line OVCAR-3 (ATCC HTB-161) and the HUVEC (ATCC CRL-1730) were used in this study. HUVECs were used as a nonmalignant control cell line to evaluate the selective cytotoxic effects of BA and PTX on ovarian cancer cells and to provide preliminary information regarding potential vascular toxicity.

OVCAR-3 cells were cultured in RPMI-1640 medium (cat. no: 11875093; Gibco, Thermo Fisher Scientific, Waltham, MA, USA), whereas HUVECs were cultured in high-glucose DMEM (cat. no: 11965092; Gibco, Thermo Fisher Scientific, Waltham, MA, USA). Both media were supplemented with 10% fetal bovine serum, 1% L-glutamine, and 1% penicillin–streptomycin. Cell cultures were maintained at 37 °C in a humidified atmosphere containing 5% CO_2_ and were passaged upon reaching 90%–95% confluence.

### 2.2. Cell viability assay

The antiproliferative effects of BA and PTX were assessed using the 3-(4,5-dimethyl-2-thiazolyl)-2,5-diphenyl tetrazolium bromide (MTT) assay (Sigma-Aldrich, St. Louis, MO, USA) [19]. A total of 5 × 10^3^ cells per well were seeded into 96-well plates and allowed to attach overnight. Subsequently, PTX was administered at concentrations ranging from 100 nM to 1 mM, whereas BA was administered at concentrations ranging from 1 mM to 40 mM. Absorbance was measured at 560 nm, and IC_50_ values were determined at 24, 48, and 72 h after treatment [20]. Cell viability as a percentage was determined for both cell lines separately. Each experiment was performed in triplicate. The concentrations used in the combination treatment experiments were selected according to the IC_50_ values obtained from the individual BA and PTX dose–response analyses. Cells in the combination treatment group were treated simultaneously with the respective IC_50_ concentrations of BA and PTX.

### 2.3. Cell cycle analysis

To evaluate whether BA and PTX induced cell cycle arrest, cells were exposed to their respective IC_50_ concentrations and subsequently stained with propidium iodide following ethanol fixation and RNase A treatment. Cells were incubated in 6-well plates at 37 °C in a humidified atmosphere containing 5% CO_2_. After 24 h, BA, PTX, and their combination were applied separately to each cell line. The cells were fixed using 70% ethanol (prechilled at 4 °C). Following RNase A treatment (200 μg/mL; Sigma Aldrich, St. Louis, MO, USA), propidium iodide (1 mg/mL; Sigma Aldrich, St. Louis, MO, USA) was added, and the cells were incubated at room temperature for 10–15 min. Cell cycle analysis was performed using an Agilent NovoCyte Penteon flow cytometer equipped with five lasers (Agilent Technologies, Santa Clara, CA, USA). Data acquisition and analysis were carried out using NovoExpress software (Agilent Technologies, Santa Clara, CA, USA) [21].

### 2.4. Apoptosis detection

To investigate the apoptotic effects of BA, PTX, and their combination in HUVEC and OVCAR-3 cells, apoptosis was evaluated using an Annexin V-FITC/PI apoptosis detection kit (BioVision, Milpitas, CA, USA). The assay was performed according to the manufacturer’s instructions. Briefly, cells seeded in 6-well plates were treated with BA, PTX, or their combination at their respective IC_50_ concentrations for 24 h. Following PBS washing, 2 μL of FITC Annexin V and 2 μL of propidium iodide (PI) were added, and the cells were incubated according to the manufacturer’s protocol. The percentage of apoptotic cells was determined by flow cytometry using a BD Biosciences flow cytometer (BD Biosciences, San Jose, CA, USA) [22].

### 2.5. Immunofluorescence analysis of autophagy-related proteins and proinflammatory cytokines

A total of 5 × 10^4^ cells were seeded onto coverslips in 24-well plates and incubated overnight to allow cell attachment. The cells were treated with PTX and/or BA for 24 h and subsequently fixed in 10% formaldehyde. Immunofluorescence staining was performed to evaluate the expression of the autophagy-related proteins Beclin-1 (1:200; Novus Biologicals, Littleton, CO, USA), LC3 (1:250; Cell Signaling Technology, Danvers, MA, USA), and p62 (1:200; Novus Biologicals, Littleton, CO, USA), as well as the proinflammatory cytokines TNF-α (1:100; Novus Biologicals, Centennial, CO, USA), IL-6 (1:100; Bioss, Woburn, MA, USA), and IL-1β (1:100; BTLab, Shanghai, China) [23]. Fluorescence intensity was quantified using ImageJ software (National Institutes of Health, Bethesda, MD, USA). Images were acquired at 400× magnification across 10 random microscopic fields.

### 2.6. Statistical analysis

Statistical analyses were performed using GraphPad Prism version 8.0.2 (GraphPad Software, San Diego, CA, USA). The normality of the data distribution was evaluated with the Shapiro–Wilk and Kolmogorov–Smirnov tests. A one-sample t-test was performed to compare the percent viability data across different drugs and dose groups. Differences among groups were analyzed using one-way analysis of variance (ANOVA) or the Kruskal–Wallis test, as appropriate. For multiple comparisons, Tukey’s test and the Dunn–Bonferroni test were applied. A p < 0.05 was considered statistically significant.

## 3. Results

### 3.1. Cell viability

**Figure 1:**
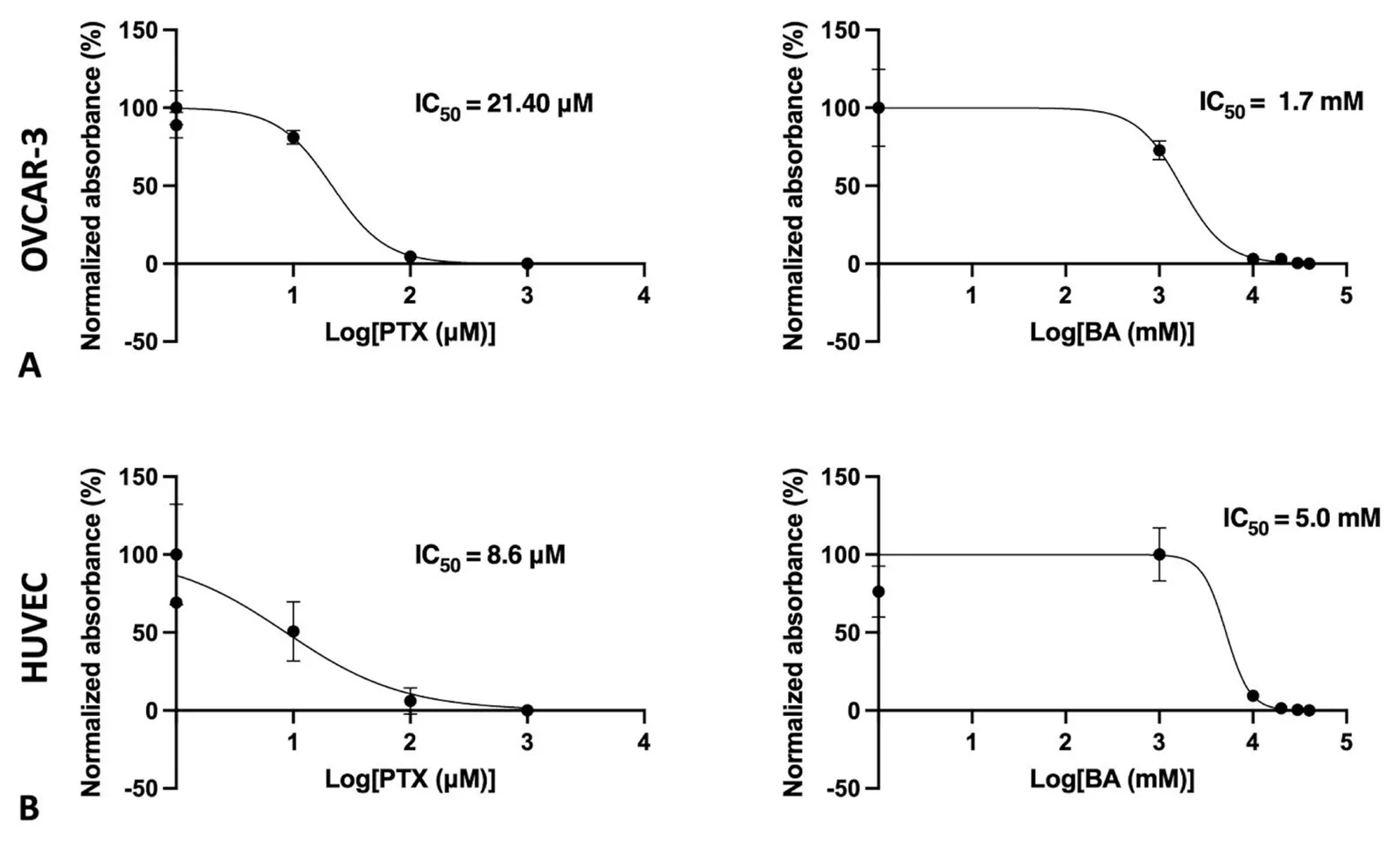
Effects of paclitaxel (PTX) and boric acid (BA) on the viability of OVCAR-3 (A) and HUVEC (B) cells after 24 h of treatment.

Cell viability assays demonstrated that BA and PTX exhibited antiproliferative effects in both OVCAR-3 ovarian cancer cells and HUVEC cells in a dose- and time-dependent manner. At 24 h after treatment, the half-maximal inhibitory concentration (IC_50_) was 21.4 μM for PTX and 1.7 mM for BA in OVCAR-3 cells. In HUVEC cells, the IC_50_ values of PTX and BA were 8.6 μM and 5.0 mM, respectively (Figure 1). These results indicate that both agents reduced cell viability in a concentration- and time-dependent manner. In addition, BA exhibited greater cytotoxicity toward OVCAR-3 cells than toward HUVEC cells.

### 3.2. Cell cycle distribution

**Figure 2:**
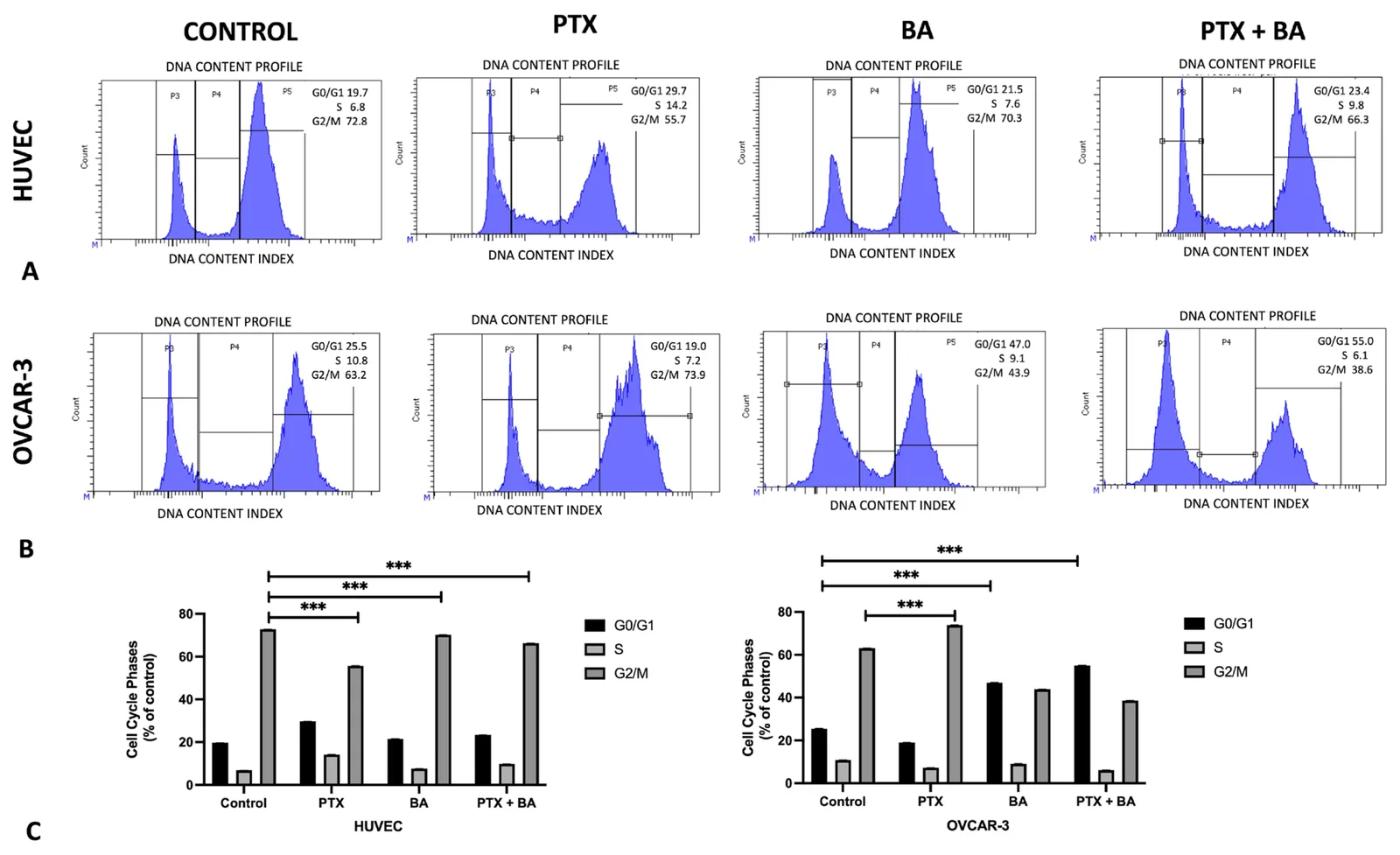
(A) Distribution of HUVEC cells among the G_0_/G_1_, S, and G_2_/M phases of the cell cycle. (B) Distribution of OVCAR-3 cells among the G_0_/G_1_, S, and G_2_/M phases of the cell cycle. (C) Quantitative analysis of cell cycle distribution in HUVEC and OVCAR-3 cells. Each experiment was performed in triplicate. ***p< 0.001 indicates a statistically significant difference.

Flow cytometric cell cycle analysis revealed that PTX, BA, and PTX + BA treatments increased the proportion of HUVEC cells in the G_2_/M phase compared with the control group, indicating treatment-induced cell cycle arrest at the G_2_/M phase (p < 0.001) (Figure 2A). OVCAR-3 cells were treated with the respective IC_50_ concentrations of BA and PTX, either individually or in combination, to evaluate their effects on cell cycle progression. BA treatment led to a statistically significant accumulation of cells in the G_0_/G_1_ phase (p < 0.001). In contrast, PTX treatment resulted in significant G_2_/M phase arrest (p < 0.001), consistent with its established mechanism of action as a microtubule-stabilizing agent. Combined treatment resulted in a significant accumulation of cells in the G_0_/G_1_ phase, accompanied by a decrease in the G_2_/M population (p < 0.001; Figure 2B). These findings demonstrate that combined treatment altered cell cycle distribution in OVCAR-3 cells, with increased accumulation in the G_0_/G_1_ phase and a reduced G_2_/M cell population (Figures 2A–2C).

### 3.3. Apoptosis

**Figure 3:**
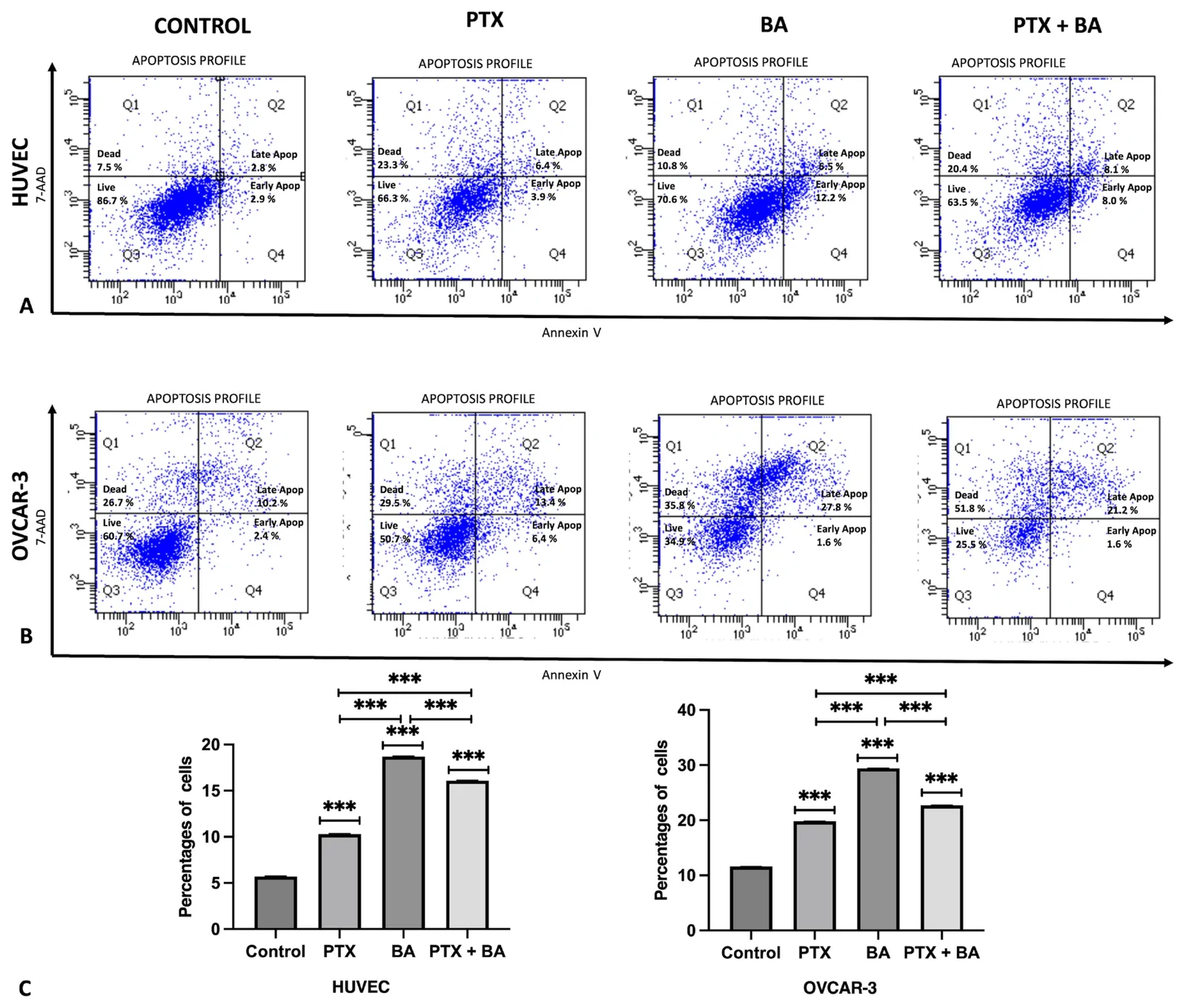
(A) Effects of PTX and/or BA on apoptotic cell death in HUVEC cells. (B) Effects of PTX and/or BA on apoptotic cell death in OVCAR-3 cells. (C) Quantitative analysis of apoptotic cell percentages in HUVEC and OVCAR-3 cells. ***p < 0.0001 indicates a statistically significant difference.

In HUVEC cells, treatment with PTX, BA, and their combination significantly increased the apoptotic cell rate compared with the control group (p < 0.001; Figure 3A). Treatment with BA and PTX, either separately or in combination, resulted in a statistically significant increase in both early and late apoptotic cell populations in OVCAR-3 ovarian cancer cells, according to flow cytometric analysis of Annexin V/PI staining (p < 0.001; Figure 3B). The combination treatment group exhibited a significantly higher apoptotic index than the PTX monotherapy group (p < 0.01; Figure 3C). These results demonstrate that combined treatment with BA and PTX was associated with a higher apoptotic index than PTX treatment alone in OVCAR-3 cells (Figure 3C).

### 3.4. Autophagy-related protein expression

**Figure 4:**
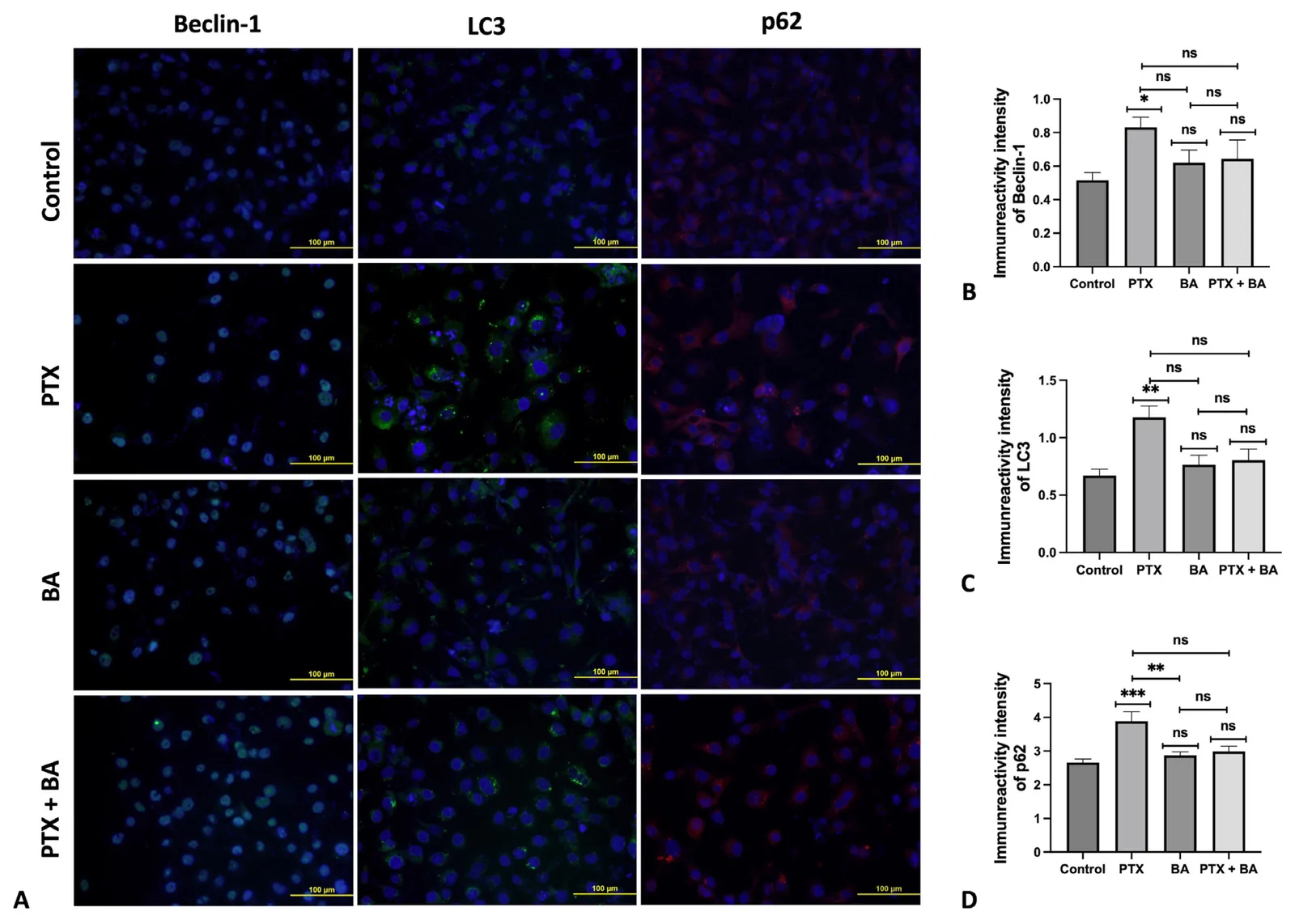
(A) Immunofluorescence staining of Beclin-1, LC3, and p62 in HUVEC cells treated with PTX and/or BA. Green fluorescence in the first column indicates Beclin-1 expression, whereas green fluorescence in the second column indicates LC3 expression. Red fluorescence in the third column indicates p62 expression. Quantitative analysis of Beclin-1 (B), LC3 (C), and p62 (D) expression. (ns: not significant; p > 0.999; *p < 0.05; ** p < 0.01; *** p < 0.001).

Immunofluorescence analysis was performed to evaluate the effects of PTX, BA, and PTX + BA treatments on autophagy-related markers in HUVEC cells (Figures 4A–4D). Compared with PTX treatment alone, combined treatment with BA and PTX showed a trend toward reduced Beclin-1 (Figure 4B), LC3 (Figure 4C), and p62 (Figure 4D) immunoreactivity in HUVEC cells; however, these differences were not statistically significant (p > 0.05).

**Figure 5:**
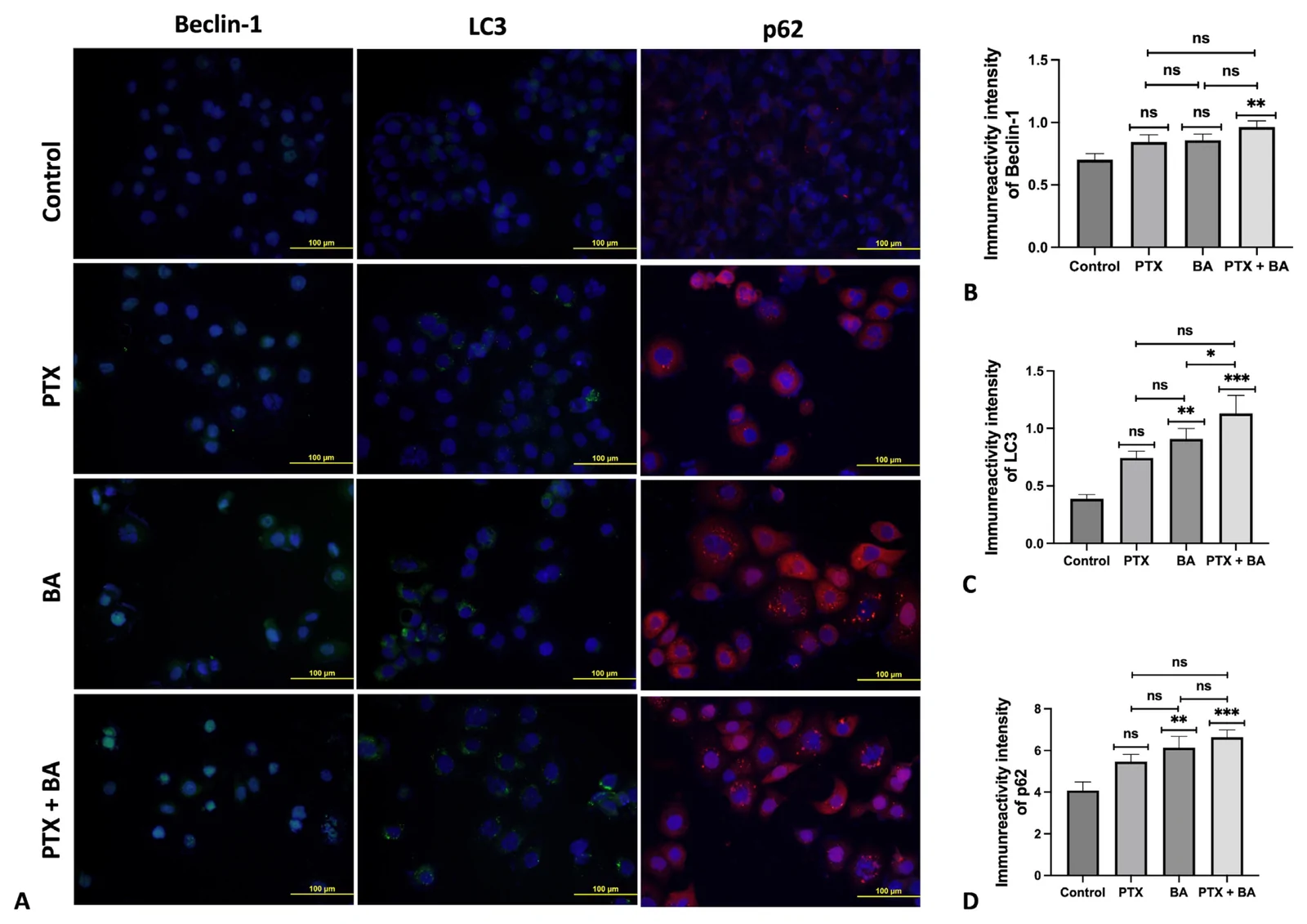
(A) Immunofluorescence staining of Beclin-1, LC3, and p62 in OVCAR-3 cells treated with PTX and/or BA. Green fluorescence in the first column indicates Beclin-1 expression, whereas green fluorescence in the second column indicates LC3 expression. Red fluorescence in the third column indicates p62 expression. Quantitative analysis of Beclin-1 (B), LC3 (C), and p62 (D) expression. (ns: not significant; p > 0.999; *p < 0.05; ** p < 0.01; *** p < 0.001).

Beclin-1, LC3, and p62 expression levels were significantly increased following treatment with BA alone or in combination with PTX in OVCAR-3 cells (Figures 5A–5D). Quantitative analysis demonstrated significant upregulation of Beclin-1, LC3, and p62 expression, with the greatest increases observed in cells treated with BA alone and in combination with PTX. Comparing these increases in protein expression to untreated control groups, quantitative analysis revealed that they were statistically significant (p < 0.05). These findings indicate that BA treatment, particularly in combination with PTX, was associated with increased expression of autophagy-related proteins in OVCAR-3 cells. In contrast, HUVEC cells did not exhibit significant changes in Beclin-1, LC3, or p62 expression following combined treatment with BA and PTX. These findings indicate differential expression of autophagy-related proteins between OVCAR-3 and HUVEC cells following treatment.

### 3.5. Proinflammatory cytokine expression

**Figure 6:**
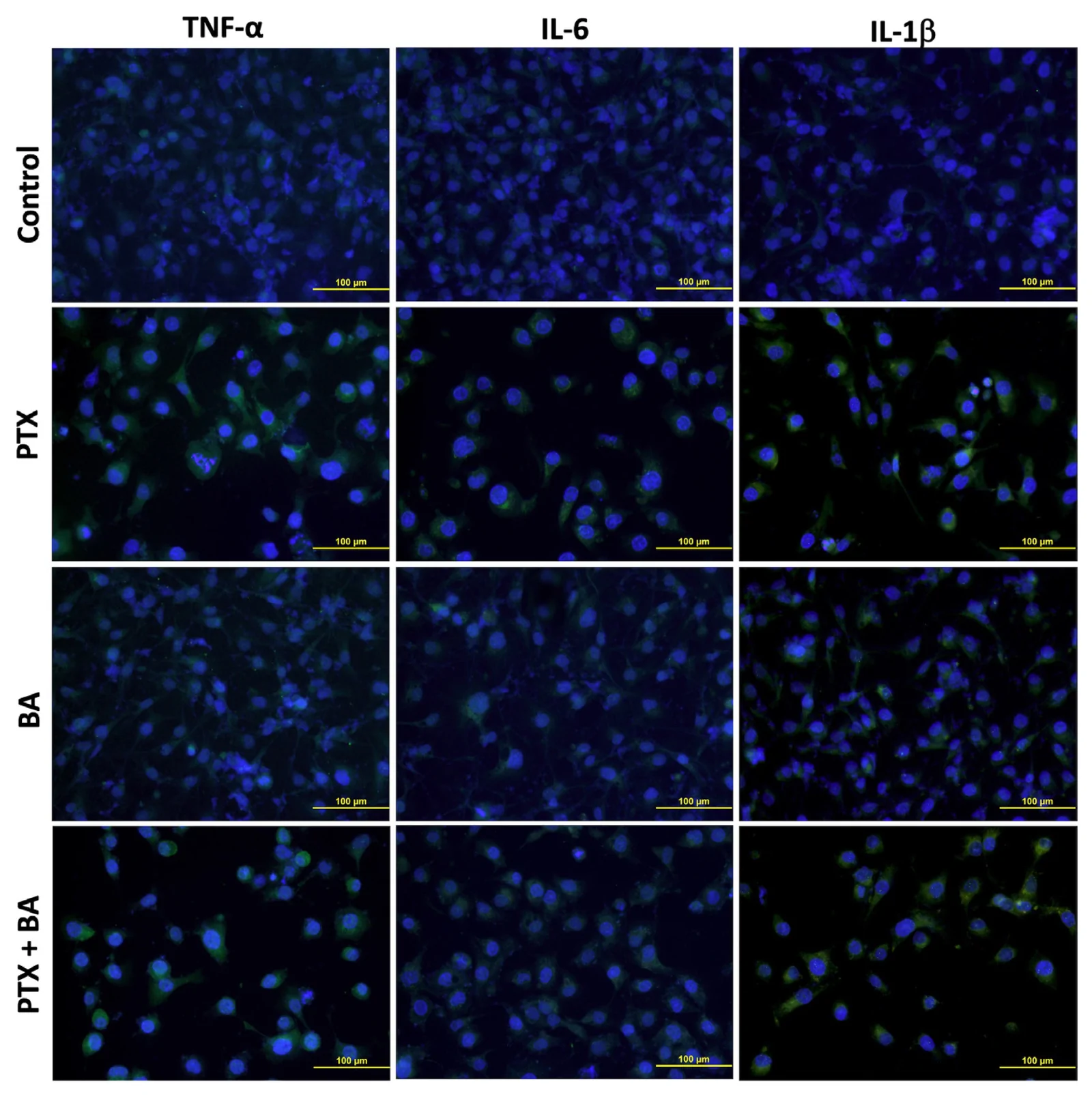
Immunofluorescence staining of TNF-α, IL-6, and IL-1β in HUVEC cells treated with PTX and/or BA. Green fluorescence in the first, second, and third columns indicates TNF-α, IL-6, and IL-1β expression, respectively.

**Figure 7:**
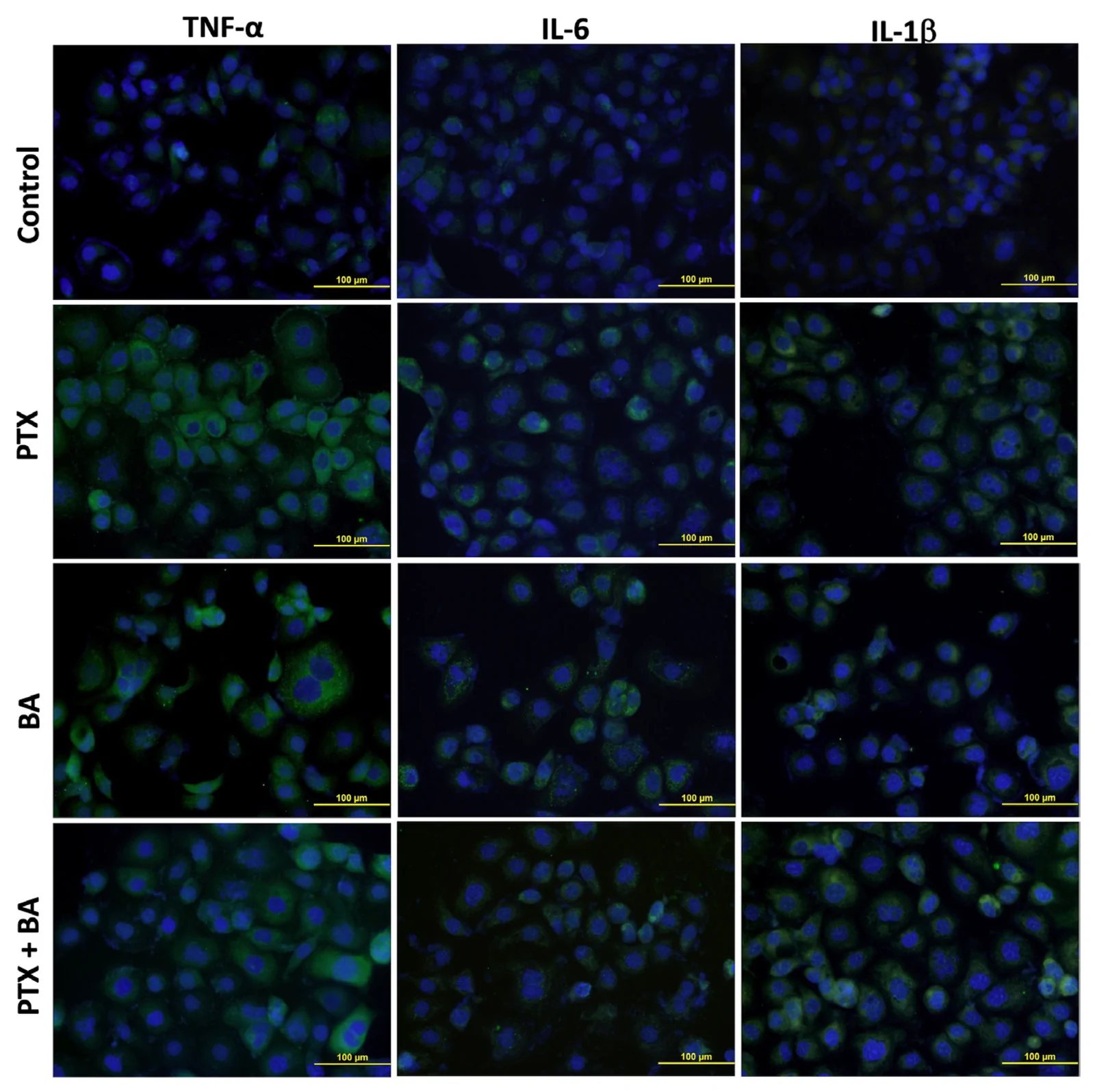
Immunofluorescence staining of TNF-α, IL-6, and IL-1β in OVCAR-3 cells treated with PTX and/or BA. Green fluorescence in the first, second, and third columns indicates TNF-α, IL-6, and IL-1β expression, respectively.

The expression levels of the proinflammatory cytokines TNF-α, IL-1β, and IL-6 were evaluated in HUVEC cells following treatment with PTX and/or BA (Figure 6). Table 1 summarizes the effects of PTX and/or BA on the expression levels of these cytokines. Immunoreactivity analysis demonstrated the expression of the proinflammatory cytokines TNF-α, IL-6, and IL-1β in OVCAR-3 cells (Figure 7). The quantitative effects of PTX, BA, and their combination on the expression levels of these cytokines are presented in Table 2. TNF-α expression was significantly higher in all treatment groups (PTX, BA, and PTX + BA) than in the untreated control group (p < 0.05), with the PTX + BA combination group exhibiting the highest expression level.

The PTX, BA, and PTX + BA groups did not, however, differ statistically significantly from one another (p > 0.999). Both PTX and BA treatments resulted in significantly increased IL-6 expression compared with the control group (p < 0.05). Although IL-6 expression was also increased in the PTX + BA group, the difference did not reach statistical significance (p > 0.999). All treatment groups exhibited higher IL-1β expression than the control group, with the PTX + BA group showing the greatest increase; however, these differences were not statistically significant (p > 0.999). Collectively, these results demonstrate differential expression of the proinflammatory cytokines TNF-α, IL-6, and IL-1β following treatment with PTX and BA in OVCAR-3 cells. In contrast, no significant changes in the expression of the evaluated proinflammatory cytokines were observed in HUVEC cells under the same experimental conditions.

## 4. Discussion

The present study investigated the effects of boric acid (BA), paclitaxel (PTX), and their combination on cell viability, cell cycle progression, apoptosis, autophagy, and inflammatory responses in chemoresistant OVCAR-3 ovarian cancer cells and nonmalignant HUVEC cells. Our findings demonstrate that BA exerts significant antiproliferative effects in OVCAR-3 cells and enhances the antitumor effects of PTX when administered in combination. These effects were associated with alterations in cell cycle distribution, increased apoptosis, modulation of autophagy-related protein expression, and changes in proinflammatory cytokine expression.

Ovarian cancer remains one of the most lethal gynecological malignancies, particularly among women older than 40 years. About 70% of cases of ovarian cancer are discovered at advanced stages (FIGO stage III or IV), which frequently limits the efficacy of traditional therapeutic approaches. Despite advances in cancer therapy, treatment resistance remains a major clinical challenge, emphasizing the need for novel therapeutic approaches [24–27]. PTX is one of the cornerstones of ovarian cancer treatment; however, resistance often limits its long-term efficacy [8,9]. Therefore, identifying agents that can enhance therapeutic responses while maintaining acceptable safety profiles remains an important goal. In this context, BA has attracted attention due to its reported antiproliferative, proapoptotic, antiinflammatory, and antioxidant properties [28,29].

Consistent with previous studies conducted in ovarian, colon, glioblastoma, and other cancer cell lines [29–31], BA significantly reduced OVCAR-3 cell viability in the present study. Moreover, BA exhibited greater cytotoxicity toward OVCAR-3 cells than toward HUVEC cells, as reflected by the lower IC_50_ value observed in OVCAR-3 cells. This finding suggests a greater cytotoxic effect of BA in OVCAR-3 cells than in HUVEC cells and supports the potential utility of BA as an adjunct to conventional anticancer therapy.

Cell cycle dysregulation is a hallmark of cancer progression, and the induction of cell cycle arrest represents a major mechanism of anticancer therapy [32]. As expected, PTX induced G_2_/M arrest, consistent with its established mechanism of action involving microtubule stabilization and mitotic blockade [8]. In contrast, BA promoted G_0_/G_1_ accumulation in OVCAR-3 cells, while combined treatment resulted in a predominant G_0_/G_1_ arrest. These findings suggest that BA may inhibit ovarian cancer cell proliferation through modulation of cell cycle progression and may complement the effects of PTX by influencing a different phase of the cell cycle. Notably, HUVEC cells did not exhibit a comparable response pattern, indicating differential effects of BA on cell cycle distribution between OVCAR-3 and HUVEC cells.

Apoptosis is one of the main mechanisms by which anticancer agents eliminate tumor cells [33,34], and fluorescently labeled Annexin V is used to identify apoptotic cells [35]. Previous studies have shown that BA induces apoptosis in various cancer models, including ovarian, colon, melanoma, and leukemia cells [11,30,36,37]. Compared with PTX treatment alone, BA treatment significantly increased the percentage of apoptotic cells. Furthermore, BA enhanced the apoptotic response when administered in combination with PTX compared with PTX treatment alone. These findings are consistent with previous reports and support the association between BA treatment, increased apoptosis, and reduced cell viability in OVCAR-3 cells.

Autophagy is a highly regulated cellular process involved in the degradation and recycling of intracellular components and plays a complex role in cancer biology [38,39]. In the present study, expression levels of Beclin-1, LC3, and p62 were significantly increased in OVCAR-3 cells following BA and BA + PTX treatments. Previous research has reported autophagy induction by BA in hepatocellular carcinoma cells [40]. Our findings extend these observations to OVCAR-3 cells and suggest that altered expression of autophagy-related proteins may contribute to the biological response elicited by BA. Importantly, no significant changes in autophagy-related protein expression were observed in HUVEC cells, indicating differential responses between OVCAR-3 and HUVEC cells. This differential response may represent a favorable characteristic of BA-based therapeutic approaches; however, further validation in additional normal cell models is warranted.

Inflammation plays a critical role in tumor development, progression, and therapeutic responses [41–43]. In the present study, BA treatment significantly increased the expression of TNF-α, IL-1β, and IL-6 in OVCAR-3 cells, indicating that BA modulates the expression of proinflammatory cytokines in this cell line. In contrast, the HUVEC cell line did not exhibit significant changes in the expression of these cytokines, indicating differential cytokine responses between OVCAR-3 and HUVEC cells. The precise contribution of these inflammatory alterations to the anticancer activity of BA requires further investigation.

In conclusion, the present study demonstrates that BA exerts antiproliferative effects in OVCAR-3 ovarian cancer cells and enhances several biological responses associated with PTX treatment, including modulation of the cell cycle, apoptosis, autophagy-related protein expression, and proinflammatory cytokine expression. Importantly, these effects were more pronounced in OVCAR-3 cells than in HUVEC cells, indicating differential responses between the two cell lines. Collectively, these findings support the potential of BA as an adjunctive agent for ovarian cancer therapy and provide a rationale for future mechanistic and in vivo studies evaluating BA-containing combination strategies.

## Figures and Tables

**Table 1:** Statistical analysis of TNF-α, IL-6, and IL-1β expression in HUVEC cells.

HUVEC	Control	PTX	BA	PTX + BA	p
TNF-α	(2.91 ± 0.36)	(3.43 ± 0.62)	(2.90 ± 0.37)	(3.35 ± 0.73)	0.060
IL-6	(1.68 ± 0.36)	(2.03 ± 0.56)	(1.73 ± 0.30)	(1.76 ± 0.43)	0.260
IL-1β	(1.97 ± 0.38)	(2.32 ± 0.61)	(1.92 ± 0.47)	(2.27 ± 0.46)	0.169

Data are presented as mean ± standard deviation. Statistical significance between groups is indicated by the p-values. A p < 0.05 was considered statistically significant.

**Table 2:** Statistical analysis of TNF-α, IL-6, and IL-1β expression in OVCAR-3 cells.

OVCAR-3	Control	PTX	BA	PTX + BA	p
TNF-α	(2.90 ± 0.80)^a^	(5.86 ± 1.65)^b^	(5.86 ± 0.93)^b^	(5.98 ± 1.03)^b^	<0.0001
IL-6	(3.95 ± 0.91)^a^	(5.48 ± 1.03)^b^	(5.63 ± 1.21)^b^	(4.59 ± 2.03)^ab^	0.0174
IL-1β	(4.12 ± 0.86)^a^	(4.52 ± 0.76)^a^	(4.57 ± 1.51)^a^	(4.66 ± 1.09)^a^	0.7062

Data are presented as mean ± standard deviation. Statistical significance between groups is indicated by the p values. Different lowercase letters within the same row indicate statistically significant differences between groups, whereas identical lowercase letters indicate no statistically significant difference. A p < 0.05 was considered statistically significant.
